# **‘**Test n Treat (TnT)’– Rapid testing and same-day, on-site treatment to reduce rates of chlamydia in sexually active further education college students: study protocol for a cluster randomised feasibility trial

**DOI:** 10.1186/s13063-018-2674-8

**Published:** 2018-06-05

**Authors:** Sarah Kerry-Barnard, Charlotte Fleming, Fiona Reid, Rachel Phillips, Vari M. Drennan, Elisabeth J. Adams, Wendy Majewska, Anjella Balendra, Emma Harding-Esch, Emma Cousins, S. Tariq Sadiq, Pippa Oakeshott

**Affiliations:** 1 Population Health Research Institute, St George’s, University of London, London, SW17ORE UK; 2 Department of Primary Care and Public Health Sciences, King’s College London, 4th Floor, Addison House, Guy’s Campus, London, SE1 1UL UK; 3 Centre for Health and Social Care Research, Kingston University and St George’s University of London, London, SW17ORE UK; 4 Aquarius Population Health Limited, 58a Highgate High Street, London, N6 5HX UK; 5 WEM Consultancy Ltd., 96 Tantallon Road, London, SW12 8DH UK; 6 Infection and Immunity, St George’s, University of London, London, SW17ORE UK

**Keywords:** Rapid chlamydia tests, Screening, Young people, Further education colleges, Test and treat, Cluster randomised, Feasibility trial

## Abstract

**Background:**

Sexually active young people attending London further education (FE) colleges have high rates of chlamydia, but screening rates are low. We will conduct a cluster randomised feasibility trial of frequent, rapid, on-site chlamydia testing and same-day treatment (Test and Treat (TnT)) in six FE colleges (with parallel qualitative and economic assessments) to assess the feasibility of conducting a future trial to investigate if TnT reduces chlamydia rates.

**Methods:**

We will recruit 80 sexually active students aged 16–24 years from public areas at each of six colleges. All participants (total *n* = 480) will be asked to provide samples (urine for males, self-taken vaginal swabs for females) and complete questionnaires on sexual lifestyle and healthcare use at baseline and after 7 months. Participants will be informed that baseline samples will not be tested for 7 months and be advised to get screened separately. Colleges will be randomly allocated to the intervention (TnT) or the control group (no TnT).

One and 4 months after recruitment, participants at each intervention college (*n* = 3) will be texted and invited for on-site chlamydia tests using the 90-min Cepheid GeneXpert system. Students with positive results will be asked to see a visiting nurse health adviser for same-day treatment and partner notification, (backed by genitourinary medicine follow-up). Participants in control colleges (*n* = 3) will receive ‘thank you’ texts 1 and 4 months after recruitment.

Seven months after recruitment, participants from both groups will be invited to complete questionnaires and provide samples for TnT. All samples will be tested, and same-day treatment offered to students with positive results.

Acceptability of TnT will be assessed by qualitative interviews of purposively sampled students (*n* = 30) and college staff (*n* = 12). We will collect data on costs of TnT and usual healthcare.

**Discussion:**

Findings will provide key values to inform feasibility, sample size and timescales of a future definitive trial of TnT in FE colleges, including:

- Recruitment rates
- TnT uptake rates
- Follow-up rates
- Prevalence of chlamydia in participants at baseline and 7 months
- Acceptability of TnT to students and college staff
- Estimate of the cost per person screened/treated in TnT versus usual care

**Trial registration:**

International Standard Randomised Controlled Trials Registry, ID: ISRCTN58038795, Registered on 31 August 2016.

**Electronic supplementary material:**

The online version of this article (10.1186/s13063-018-2674-8) contains supplementary material, which is available to authorized users.

## Background

There are high rates of sexually transmitted infections (STIs) in ethnically diverse, sexually active students aged 16–24 years attending London further education (FE) colleges [1–4], with around 8% testing positive for *Chlamydia trachomatis*. However, uptake of chlamydia screening remains low: below 30% annually in 16–24-year-olds in England [4, 5]. Although chlamydia primarily affects young people, the consequences of infection such as infertility, chronic pelvic pain or epididymitis can last a lifetime. It is estimated that 10–16% of women with untreated chlamydia will develop clinical pelvic inflammatory disease of whom 8% will have an ectopic pregnancy and 11% will suffer from tubal-factor infertility [6]. The cost of chlamydia to the NHS is estimated to be over £100 million each year.

Barriers to reducing chlamydia rates include low uptake of testing by those most at risk [5, 7] (such as sexually active teenagers, people from ethnic minorities and people who are socioeconomically deprived), and long delays in receiving a positive diagnosis or attending for treatment. Introducing rapid, on-the-spot chlamydia tests and treatment into the community could make it easier for young people to get tested and treated faster, and before they can pass on their infection. It might also prevent complications [8, 9]. These novel tests can have 99% sensitivity and 99.4% specificity [10], and studies have demonstrated their feasibility in remote communities [11]. However, there have been no UK trials of rapid STI tests and same-day, on-site treatment (Test and Treat (TnT)) in non-healthcare settings.

We will use a test that checks for gonorrhoea as well as chlamydia as this would likely be included in a real-life roll out. However, as people diagnosed with gonorrhoea are best managed by a sexual health clinic they will not receive on-site treatment. Hence, TnT will be just for chlamydia.

Our cluster randomised feasibility trial aims:

1. To assess the feasibility of conducting a trial of TnT in FE colleges, and obtain estimates of key values to inform sample size estimates and timescales for a definitive trial
2. To explore the acceptability of TnT through a qualitative evaluation
3. To estimate the cost per person tested and treated in TnT versus usual care

## Design

**Fig. 1:**
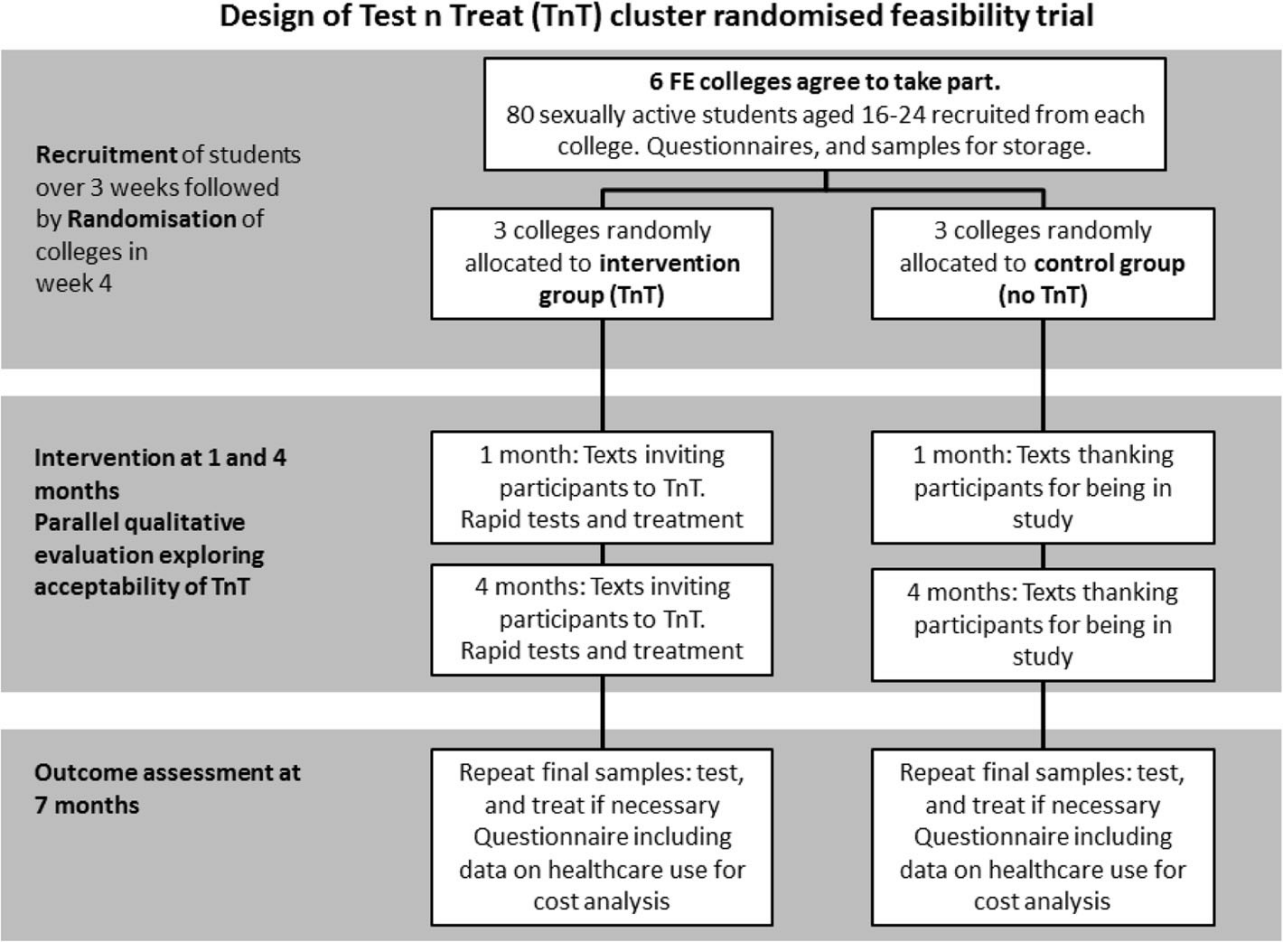
Shows the design of the Test n Treat (TnT) cluster randomised feasibility trial

**Fig. 2:**
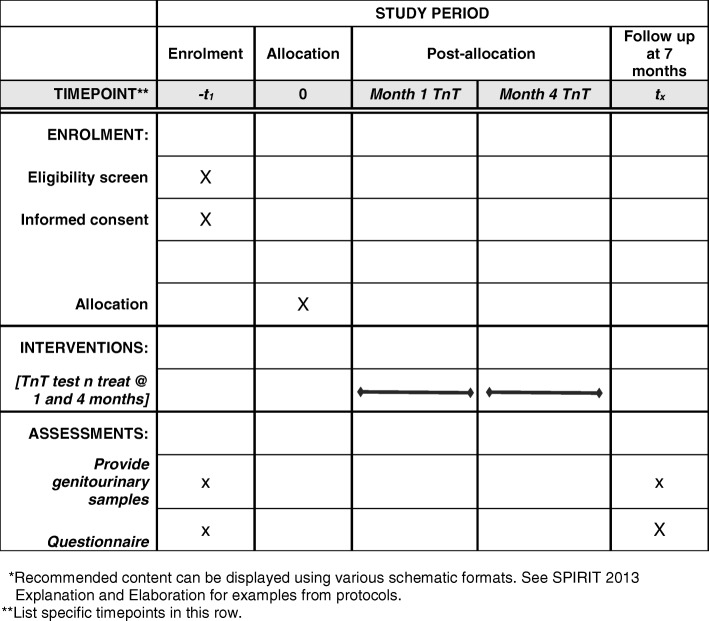
Standard Protocol Items: Recommendations for Interventional Trials (SPIRIT) Figure showing the schedule of enrolment, interventions, and assessments for the Test n Treat (TnT) chlamydia screening feasibility trial

Cluster randomised controlled feasibility trial over 7 months with parallel qualitative and economic assessments (Fig. 1 and Additional file 1: Standard Protocol Items: Recommendations for Interventional Trials (SPIRIT) 2013 Checklist (Fig. 2)). The outcome is within one academic year to optimise follow-up.

### Setting

Six ethnically diverse FE colleges in London FE colleges take students from the age of 16 years and teach both academic subjects and vocational courses such as plumbing and hairdressing. As previously [3] we will first obtain agreement from staff and student bodies.

### Participants

At each site during 2 days, 80 consecutive sexually active students (total 480 students across all sites) aged 16–24 years will be recruited. We previously found that such students are a high-risk group: 43% report two or more sexual partners in the past year, 34% smoke cigarettes, 50% are teenagers and 30% are from black ethnic minority groups [3].

#### Exclusion criteria

Students who have never had penetrative sexual intercourse; students with severe learning disability.

### Recruitment and consent (September to October 2016)

Research assistants will approach students in common room areas [3, 12]. The students will be asked if they are willing to help with research on sexual health. Students aged 16–24 will be invited to come to the study table where recruiters will explain that as the study is about chlamydia and sexually transmitted infections, only students who have had penetrative sexual intercourse should consider taking part. Those who are interested will be given a patient information sheet and consent form to read and be encouraged to ask questions.

The information sheet will explain that participants will be asked to complete short confidential electronic questionnaires on sexual health using a tablet computer, and to provide samples on this day and again after 7 months (urine for males and self-taken vaginal swabs for females). In half the colleges, students will also be asked to provide samples for rapid testing for chlamydia and gonorrhoea after 1 and 4 months, and those found to be infected with chlamydia will be given same-day, on-site treatment. Some students may also be invited to take part in interviews [13].

Research assistants will ensure that all participants understand that samples provided at recruitment will not be tested for 7 months and it is the student’s responsibility to get tested separately [3] (e.g. at a sexual health clinic or at a general practice) if they are allocated to the control group or if they want to be tested for other STIs such as HIV.

These stored baseline samples are for three reasons:

1. To emulate the processes of a future definitive trial
2. To ensure that all participants know how simple it is to provide samples for testing
3. To measure baseline chlamydia prevalence at each site in order to refine the intra-class correlation coefficient (ICC) for sample size calculations

Students who agree to take part will be asked to sign a consent form and to provide full contact details including mobile number and email for follow-up and text reminders [3]. They will be asked for consent to obtain their NHS numbers and to allow the researchers to access their GP, hospital, and genitourinary medicine (GUM) clinic records [14].

### Honoraria for participants

Honoraria will be provided both to facilitate recruitment [15, 16] and to encourage participants to return repeat samples at the end of the trial. (We have ethical approval for honoraria and have shown them to be very effective [16]). Participants will be given £5 when they return completed sample packs at recruitment, and £10 at the final 7-month follow-up. Participants in the qualitative interviews will be given £10 for their time. However, participants in intervention colleges will not be given honoraria to attend for TnT after 1 and 4 months as this would not happen were the intervention to be rolled out in future.

### Data collection at baseline (September to October 2016)

During  recruitment, participants will be asked to complete a confidential baseline questionnaire using a tablet computer. Questions will include date of birth, self-assigned ethnicity, smoking, alcohol, age at sexual debut, condom use, contraception, number of sexual partners in the previous 12 months, when they last had sex with a new partner, recent STI testing and treatment, history of STI, and genitourinary symptoms in the past 6 months [3]. All participants will also be asked to provide samples in the nearest toilet.

### Randomisation

Randomisation will take place once recruitment is completed and baseline data collected for all colleges.

Using a computer programme, colleges will be randomly allocated into the intervention (TnT) or the control (no TnT) by the trial statisticians RP and FR. The randomisation will be constrained to ensure that three colleges are allocated to each group.

### Intervention TnT colleges – rapid chlamydia/gonorrhoea testing and same-day chlamydia treatment (1 and 4 months after recruitment)

In November 2016, 1 month after recruitment, each intervention campus will be visited on two consecutive days by the TnT team. These will be the same days of the week as at recruitment to optimise student attendance. The 80 participating students in each campus will be texted and invited to come for on-site rapid chlamydia/gonorrhoea testing and same-day treatment for chlamydia. As at recruitment they will be invited to complete a questionnaire and provide a sample, but this time the sample will be tested immediately on site using the Cepheid GeneXpert system which takes 90 min. Participants will be given a card containing information about the local GUM/sexual health clinic, a link to the Brook sexual health website: https://www.brook.org.uk, and TnT study contact details.

Negative results will be texted. Participants with positive results will be telephoned by the nurse health adviser and invited to see her in the college nurse’s room for confidential same-day treatment if positive for chlamydia, partner notification, advice, and follow-up. Infected students will be asked to bring any sexual partners who attend the college so they can also be tested and treated. (In a survey in 2014, 9% of 103 students said they had a sexual partner at the same FE college.) Students who are positive for gonorrhoea will be asked to attend St George’s NHS Trust GUM clinic for further testing and review by a clinician.

All participants in the three intervention colleges will be invited to provide repeat samples for on-site TnT 4 months after recruitment (i.e. the next term January–February 2017).

### Control ‘usual care’ colleges (1 and 4 months after recruitment)

Participants from the three control colleges will not get TnT but will receive texts 1 and 4 months after recruitment thanking them for being in the study.

### Data collection at 7-month follow-up (April–May 2017)

All participants will be asked to provide samples for TnT and to complete questionnaires at college at 7 months. Follow-up questionnaires will include additional questions about STI testing and treatment, oral sex, vaccination against human papillomavirus (HPV), and use of healthcare services for sexual health since recruitment date, including attendances at general practice, sexual health or hospital clinics, hospital admissions, and drug treatment [3, 17].

When we invite them to the outcome assessment we will send participants a link to an additional consent form and information sheet explaining that we will be asking them to provide optional mouthwash samples (for future testing for HPV and chlamydia/gonorrhoea) as well as genitourinary samples. We will also put this information on the study webpage. For those who attend we will provide paper copies of the additional consent form and information sheet and answer any questions. We will explain that providing the mouthwash sample is optional and for research purposes only and, that as chlamydia/gonorrhoea and HPV tests are not validated on these samples, we will not feedback results. Those who agree will be asked to sign the additional consent form.

Testing at 7 months is required in the proposed full trial in order to calculate the main outcome (prevalence of chlamydia), and is included here to test the feasibility of collecting these data. It will also help inform estimates of effect size for sample size calculations. Treatment of those diagnosed with infection at 7 months will also be offered. This is not part of the assessed intervention, but is offered to enhance testing uptake at this time, and participation in general among the control group. In addition, at the end of the study stored baseline samples will be tested using standard tests, and students with positive results will be contacted by the health adviser [3].

### Masking

Recruitment of colleges and participants will be conducted prior to group allocation^3,19^. Therefore, the baseline data collection from students will be blind to treatment group. By the time of the first TnT intervention 1 month after baseline, participants and research assistants will no longer be blinded.

### Qualitative evaluation exploring the acceptability of TnT

A research assistant will conduct semi-structured interviews [13] with a purposive sample of male and female college staff and students to investigate views on the acceptability of TnT including barriers and facilitators to uptake and possible harms.

1. *College staff (n = 12)*

We will interview teaching and student welfare staff and explore opinions of the acceptability of the intervention, barriers, facilitators, harms, and challenges to reaching certain subgroups such as male teenagers from ethnic minorities. We will also explore views on the trial methodology and suggested improvements.

2. *Students (n = 30)*

These interviews will explore opinions on acceptability, barriers, and facilitators to uptake of TnT, and views of potential harms of on-site rapid tests and treatment. We will also seek views as to the trial methodology and suggested improvements.

Interviews will focus on three distinct groups:

1. Students who declined to participate in the trial (*n* = 10) who agree to be interviewed to explore factors influencing their decision

2. Participants (*n* = 10) in intervention sites who used TnT

3. Participants (*n* = 10) in intervention sites who were recruited but did not use TnT

Interviews will be digitally recorded with permission, transcribed and thematically analysed.

### Health economic analysis

We will estimate the cost per person screened and treated of implementing TnT in FE colleges compared with usual care (no TnT), and the incremental cost per chlamydia infection averted. Costs will be classified as solely research; capital set-up costs; or on-going running costs. We will also assess the feasibility of obtaining questionnaire data on healthcare use during the study period. Based on what participants report in their 7-month questionnaires about healthcare setting attended for chlamydia-related problems, we will explore the feasibility of estimating the health resources used in both arms of the study using published tariff costs for GUM, hospital inpatient and outpatient visits, and GP attendance [17]. We will explore the marginal costs of offering TnT at 1 and 4 months compared to no test. Results will inform the cost of implementing TnT in a definitive trial.

### Main outcomes

1. Key values to inform feasibility, sample size, and timescales of a full trial of TnT in FE colleges:
  - Recruitment rates:
    Recruitment rate: colleges and students
    Time taken to recruit 80 participants at each site
    Age, gender, and ethnicity of students recruited versus not recruited [16]
  - Testing and treatment uptake rates (1 and 4 months after recruitment):
    Testing and treatment uptake rates, in intervention sites only
    Time from provision of sample to treatment of chlamydia positives
  - Follow-up rates (at 7 months):
    Percentage providing samples at all sites
    Percentage completing final questionnaires (including data on healthcare usage)
  - Prevalence of chlamydia in participants at each site at baseline and at 7 months. Chlamydia prevalence at baseline will enable us to refine the ICC, and prevalence at 7 months to refine the estimated effect size, both required for sample size calculations for the substantive trial
2. A perspective on the acceptability of TnT in FE colleges, emerging from qualitative interviews with purposively sampled students and college staff
3. Health economic analysis (as described above)

Estimate of the cost per person screened/treated in TnT versus usual care.

### Sample size and statistical analysis

Assuming a 30% recruitment rate [12], 1600 students will be approached to recruit 480 overall (80 per site across six sites, three intervention, and three control). Estimates of testing uptake at 1 and 4 months (intervention sites only) will be based on 240 students, and at 7 months will be based on 480 students (all sites).

Teare et al. [18] recommend that 60 to 100 subjects is sufficient to estimate an event rate with acceptable precision in a feasibility study. Prevalence of chlamydia at baseline will be estimated separately for each of the six sites (80 students per site), and these prevalences will be used to inform the intraclass correlation coefficient (ICC), required for the sample size calculation for the main study. From our previous research involving 11 colleges [3], the ICC was estimated to be 0.005 (95% confidence interval (CI) − 0.013 to 0.026). Adding data from another six colleges will improve the precision of this ICC, reducing the width of the confidence interval by around 20%.

Assuming 70% followed up at 7 months (with £10 honoraria), final estimates of chlamydia prevalence would be based on 168 students in each of the intervention and control groups. The study is not powered to find a statistically significant difference, but this may provide useful information on possible effect size to inform future sample size calculations.

### Statistical analysis

#### Summary of baseline data and flow of patients

A Consolidated Standards of Reporting Trials (CONSORT) flow diagram will be produced to show the number of students recruited, the numbers attending at 7-month final follow-up, and for those not attending the reasons (where available) for non-attendance including lost-to-follow-up and withdrawal. Baseline descriptions of students recruited to the study will be presented by treatment arm: including means and standard deviation or numbers and proportions as appropriate. This will be repeated to compare the baseline characteristics of those in the intervention arm returning for TnT at 1 and 4 months compared to those who did not return. Where available reasons for non-attendance at the 1- and 4-month visits will be summarised. Summary characteristics of participants at the FE colleges will also be summarised by treatment arm. No statistical significance testing will be performed.

#### Main outcomes analysis

Recruitment rates (colleges and students) will be calculated as proportions with corresponding 95% CIs. This will include the proportion of colleges participating in the study of the total number of colleges asked to participate, and, where available, the proportion of students who were eligible of the total number of students who were assessed for eligibility. The proportion of students tested, who obtain a positive test result and receive treatment, will be calculated at 1 and 4 months for the intervention group and at 7 months for both the intervention and the control groups, and corresponding 95% CIs will be presented.

The time taken to recruit students will be summarised by means and standard deviations or medians and inter-quartile ranges as appropriate, for each site and overall. At intervention colleges we will summarise the time from providing a sample to treatment for students with a positive chlamydia test at 1 and 4 months with means and standard deviations or medians and inter-quartile ranges as appropriate. All confidence intervals will be two-sided and will be at the 95% level. A detailed statistical analysis plan has been developed and approved by the Trial Steering Committee [19].

## Discussion

To our knowledge this will be the first UK cluster randomised study exploring the feasibility of rapid chlamydia tests and same-day, on-site treatment in the community. If the findings lead to a main trial which shows that TnT is acceptable, cost-effective and reduces chlamydia rates, implementing TnT in the community might improve the sexual health of many hard-to-reach young people.

### Trial status

We are recruiting participants.

## Additional file

Additional file 1: Standard Protocol Items: Recommendations for Interventional Trials (SPIRIT) 2013 Checklist: recommended items to address in a clinical trial protocol and related documents. (DOC 121 kb)

## Abbreviations

FE colleges: Further education colleges
GUM: Genitourinary medicine
HPV: Human papillomavirus
ICC: Intraclass correlation coefficient
STI: Sexually transmitted infection
TnT: Test n Treat
